# Elevated Blood Ammonia Level Is a Potential Biological Risk Factor of Behavioral Disorders in Prisoners

**DOI:** 10.1155/2015/797862

**Published:** 2015-09-17

**Authors:** Yunfeng Duan, Xiaoli Wu, Shan Liang, Feng Jin

**Affiliations:** ^1^Key Laboratory of Mental Health, Institute of Psychology, Chinese Academy of Sciences, No. 16, Lincui Road, Chaoyang District, Beijing 100101, China; ^2^University of Chinese Academy of Sciences, Beijing 100101, China

## Abstract

Hydrothion (H_2_S) and ammonia (NH_3_) can be toxic for the human central nervous system and cause psychological disturbances and behavioral disorders. In order to evaluate the association between the two potential toxicants and mental health, in this study, we compare a male prisoner and control population. Forty-nine male prisoners and 52 control volunteers took part in the study. An aggressive behavior assessment, the Self-Rating Depression Scale (SDS), and the State-Trait Anxiety Inventory (STAI) were used to characterize the participants' mental health status. Venous blood was collected for detection of H_2_S and NH_3_. The results indicated that blood NH_3_ was significantly higher in male prisoners than in controls. However, blood H_2_S was significantly lower. Blood NH_3_ was also significantly and positively correlated with prisoners. In the multivariate adjusted models, after controlling for age, education, marital status, and BMI, we found a positive association between NH_3_ and prisoners, but not blood H_2_S. While the functions of the two toxicants were quite different, blood NH_3_ may be a potential biological risk factor for behavioral disorders and blood H_2_S showed neuroprotection. Additionally, the impact of other factors such as diet and gut bacteria should be considered when evaluating risk for behavioral disorders.

## 1. Introduction

Ammonia (NH_3_) and hydrothion (hydrogen sulphide, H_2_S) have long been known for their poisoning effect to human central nervous system and may cause psychological disturbances and behavioral disorders. In the brain, NH_3_ supplied by the blood stream exerts its neurotoxic effects by inducing astrocyte swelling and triggering a reaction cascade [1–3]. Previous studies have shown that patients with shock [4] and hypoxia [5] have increased levels of blood NH_3_. NH_3_ is also implicated in hepatic encephalopathy, whereby its toxicity is mediated by glutamine, which is metabolized from NH_3_ in the brain [6]. NH_3_ widens junctions in the blood-brain barrier, allowing not only pathogens, but also small molecules like glutamate along with neutrophils and water to penetrate the barrier [7]. Hyperammonemia may also result in irreversible brain damage [8]. Exposure to high level of NH_3_ can alter several amino acid pathways and neurotransmitter systems, cerebral energy metabolism, nitric oxide synthesis, oxidative stress, and signal transduction pathways in the brain [9].

H_2_S is a neurotoxin that can cause neuronal death [10]. However, as a gasotransmitter, it is endogenously produced in the brain targets multiple molecules that affect neuronal viability in the mammalian brain [11]. In the central nervous system (CNS), it is considered to be a physiological mediator and appears to participate in cognition, memory, regulation of the cardiopulmonary functions, and neuroprotection [12]. In the peripheral nervous system, evidence suggests that H_2_S may be involved in autonomic control of the cardiopulmonary and gastrointestinal functions as well as pain and inflammation [13].

One study found that the plasma level of H_2_S was markedly lower in patients with depression [14]. It is also shown that H_2_S synthesis in the brain is severely reduced in Alzheimer's disease (AD) patients [15, 16], and plasma H_2_S levels are negatively correlated with the severity of AD [17]. Further, inhaled H_2_S was able to prevent neurodegeneration in a mouse model of Parkinson's disease, another incurable, chronic neurodegenerative disorder [18]. All these results indicate that H_2_S plays a role in neuron protection and that lower level of H_2_S is harmful to normal brain function.

Additionally, H_2_S-related sulfur metabolites are also involved in some mental disorders. Autism spectrum disorder (ASD) is associated with a substantially reduced level of plasma sulfate but increased urinary excretion of sulphate, sulphite, and thiosulphate [19]. Parents of autism children also are subject to impairment in sulfur metabolism [20–22]. Further, ASD is also associated with excessive exposure to nitric oxide, NH_3_, and glutamate in the CNS [23].

It was estimated that approximately 100 million people in China and 450 million people worldwide currently suffer from mental health problems [24]. Roughly a quarter of people will experience some sort of mental disorder in their lifetime. Prisoners are prone to having mental health problems than the general population, such as depression, anxiety, violence, and even mental disorders. More than 60% of male prisoners are estimated to have serious mental problems around the world [25]. In the USA, there are ten times more prison inmates with mental problems than all the patients in state hospitals [26]. In the UK, it is estimated that about nine out of ten prisoners have mental problems while seven out of ten have at least two types of mental disorders [27]. Additionally, male prisoners are more likely to have mental problems.

There is lack of information regarding a possible association between NH_3_ and H_2_S and mental problems in prisoners. In order to examine whether NH_3_ or H_2_S is potential biological risk factor of mental problems, in this study, we use questionnaires and lab tests to investigate the correlation between blood NH_3_/H_2_S levels and mental problems by contrasting adult male prisoners and healthy controls.

## 2. Materials and Methods

We recruited 49 male prisoners and 52 male controls from Northern China. Control participants were recruited from the area where the penitentiary is located using a systematic random sampling method. All participants were medically healthy. The jail sentences were less than one year for 82.8% of the prisoners and more than one year for the rest 17.2% (*M*_time_ = 3.38 months, SD = 1.52). The crimes committed by these prisoners include both aggressive offenses (85%) such as murder, forcible rape, robbery, and aggravated assault and nonaggressive offenses (15%), such as burglary, national property damage, and drug-related crimes.

The SDS and STAI were used to measure depression and anxiety, respectively. In particular, aggression was evaluated using an aggressive behavior assessment (aggressivity; bullying; fighting; breaking rules; and quarrelling and the number of times in the previous one month). The assessment was rated by the correctional officers, friends, or close acquaintances, but not family members, of the participants.

To minimize the possible effects from medication, blood samples were collected only from those who were free from any drugs for at least four weeks. Blood collection was performed in the morning between 09:00 a.m. and 12:00 p.m., soon after the participants had completed the questionnaires. Venus blood was collected via venipuncture in tubes containing heparin sodium and ethylenediaminetetraacetic acid (EDTA). Whole blood NH_3_ levels were measured using chemical assay kits on a VITROS-350 dry chemical analyzer (Johnson & Johnson Diagnostics, United States). Whole blood H_2_S levels were determined using a Human H_2_S ELISA Kit (Product number ABIN511370, Antibodies Online).

The data followed normal distributions (Kolmogorov-Smirnov *Z* = 0.92, *p* = 0.36); thus these data were used without transformation. Demographic data and psychiatric scales were used to adjust for potential confounding factors or effect modifiers.

Independent sample *t*-tests were performed to compare the mean values of continuous variables between the groups. Chi-square tests were applied to compare categorical variables between the groups. A logistic regression analysis was performed to explore the association between blood biochemical variables (H_2_S and NH_3_ levels) and behavioral problems. In the first model, the blood biochemical variables were used as the independent variables, followed by adjustments for age, education, marital status, BMI, smoking, and drinking. In the second model, we further get psychiatric scales included for adjustment. A forward:LR (Likelihood Ratio) method was used in both models. All statistical analyses were conducted using IBM SPSS Statistics 22.0 and Microsoft Office 2007, with statistical significance defined as an alpha level of 0.05. Two-tailed probabilities were applied throughout.

This study protocol was approved by the Institutional Ethics Committee, and a written informed consent was obtained from all participants after signing before data collection.

## 3. Results and Discussion

The demographic variables did not differ significantly between the prisoner and control groups (Table 1). Among the psychological and biological variables, depression scores, state and trait anxiety levels, and aggressive behaviors were significantly higher in male prisoners than in the controls (Table 2). The blood NH_3_ levels were significantly higher in the prisoners than in the controls, whereas the blood H_2_S levels were significantly lower in the prisoners.

In the first model, an inverse association was found between H_2_S and prisoners (OR: 0.035; 95% CI: 0.001–1.133, *p* > 0.05), although it did not reach significance. In contrast, there was a significant positive correlation between NH_3_ and prisoners (OR: 1.111; 95% CI: 1.055–1.171, *p* < 0.001). A positive association between NH_3_ and prisoners (OR: 1.189; 95% CI: 1.072–1.319, *p* < 0.001) was found even in the multivariate adjusted model, which controlled for potential confounders (age, education, marital status, and BMI). In the second model, after controlling for potential confounders (age, education, marital status, BMI, SDS, STAI-S, and STAI-T), we only found a significant positive correlation between NH_3_ and prisoners (OR: 0.730; 95% CI: 0.576–0.924, *p* < 0.01). It can thus be concluded that blood NH_3_, but not blood H_2_S, successfully predicted each participant's group.

From the study, blood NH_3_, but not H_2_S, was an independent prognostic factor for behavioral disorder, and thus these biomarkers may be useful in predicting poor mental status for screening or intervention purposes.

Elevated NH_3_ is often detected in intrinsic liver disease but can also be caused by abnormal catabolic state (e.g., excess of amino acids degradation) or reduced clearance [28]. Since all the participants claimed to be healthy and should not have liver disease, the elevated NH_3_ should instead result from food digestion, reduced clearance, or both.

Mounting evidence has shown that diet can influence our feelings of anxiety [24], depression [29–31], anger [32], and stress [33] and even lead to psychological conditions such as attention deficit hyperactivity disorder [34]. In particular, metabolism of protein and amino acids, the primary nutritional intakes from diets, is important to the maintenance of mental health because proteins are degraded to amino acids and then to H_2_S and NH_3_. Meat is a valuable source of protein and fat, which are beneficial to health in appropriate amounts but harmful in excess. It is suggested that eating meat as part of an unhealthy diet can lead to mental illness and psychological distress [35–37]. One recent study reported that stress levels were inversely related to meat consumption after adjusting for sex, age, smoking, and physical activity [33]. Additionally, studies in adults have shown that diets high in processed foods, like chocolate, refined grains, and processed meat, may increase the likelihood of depression and both trait and state anxiety levels were significantly related to the consumption of processed foods [38]. Another study found that males were more likely to have diets high in animal products and that unhealthy eating patterns (including meat and snack foods) were associated with higher risk for behavioral disorders like depression and anxiety [39].

However, lowering meat consumption does not necessarily reduce NH_3_ production, but the dietary fiber (high in fruits and vegetables) does [40].

Some studies have reported that women who consumed less than the recommended amount of red meat were more likely to be diagnosed with a depressive or anxiety disorder than those who consumed the recommended amount [41]. Another study showed that lower levels of stress were linked to a diet high in unsaturated oils, grains, fruits, vegetables, meat, and dairy products [33]. Therefore, diets containing fruits, vegetables, and nuts are considered an important protective factor against psychological illness, promoting or maintaining a healthy mental state [30, 42–44].

Although many studies have indicated a relationship between diet and mental health, the biological mechanisms involved are still not well understood. It is suggested that NH_3_ and H_2_S both were affected by the gut microbiota. Certain bacteria species residing the mouth and gastrointestinal tract can generate H_2_S during the metabolism of sulfhydryl-containing amino acids (e.g., cysteine) from diet [45]. The* Desulfovibrio* species in the gut can also metabolize sulfate to H_2_S [46]. Excessive NH_3_ produced by yeast in the gut has also been linked to chronic inflammation and autism [47, 48].

In this study, we used an exclusively male population; a report showed that men typically consume more processed foods and meat but less vegetables than do women [38, 49]. Depressive mood and aggression are also related to amino acid metabolism, and dietary tryptophan depletion has been shown to increase aggression in healthy men, likely by decreasing brain serotonin [50, 51]. The increased NH_3_ might be caused by diet and we hypothesized that an unhealthy diet would be associated with a higher likelihood of depressive and anxiety disorders and a greater prevalence of psychological symptoms.

## 4. Conclusions

In sum, blood NH_3_ could act as a potential biological marker of behavioral disorders. However, further studies are needed to address how blood NH_3_ regulates brain function and contributes to the development of behavioral disorders. In particular, it should be taken into consideration whether the high blood NH_3_ levels result from increased production or decreased clearance, as well as the liver health status of participants. Additionally, production of NH_3_ and H_2_S from endogenous or exogenous sources, that is, host or gut microbiota, should be distinguished. Moreover, since NH_3_ and H_2_S carry distinct functions, the impact from factors such as diet and gut bacteria should also be considered to explore the relationship between H_2_S and behavioral disorders.

## Figures and Tables

**Table 1:** Demographic summary of the participants.

	Prisoner (*n* = 49)	Control (*n* = 52)	*t*/*χ* ^2^	*p* value
Age	33.20 (1.27)	30.13 (1.15)	1.8	0.080
Marital status				
Single	67.40%	51.10%	1.58^*∗*^	0.053
Married	25.60%	46.70%		
Others	7%	12.80%		
Education				
Primary school	45%	30.50%	−1.79^*∗*^	0.056
High school	45.50%	57.70%		
Higher	9.10%	12.80%		
BMI	22.90 (2.8)	24.20 (3.80)	−1.87	0.060
Smokers	75%	57.80%	2.95^*∗*^	0.086
Drinkers	54.40%	46.70%	0.55^*∗*^	0.457

^*∗*^Chi-square test result.

**Table 2:** Independent *t*-test for psychological and biological test results.

	Prisoners	Controls	*t*	*p* value
	*N* = 49 Mean (SD)	*N* = 52 Mean (SD)		
STAI-S	2.4 (0.5)	1.9 (0.4)	4.61	0.000
STAI-T	2.5 (0.4)	2.0 (0.4)	2.54	0.013
SDS	42.5 (7.5)	37.4 (9.0)	2.85	0.006
Aggression	10.5 (2.6)	7.8 (1.1)	6.58	0.000
H_2_S (pg/mL)	0.4 (0.2)	0.5 (0.15)	−2.36	0.021
NH_3_ (*µ*mol/L)	42.2 (10.1)	30.2 (13.8)	4.70	0.000
